# The environmental impact of the sugar industry waste in Sudan

**DOI:** 10.1007/s10661-023-11401-8

**Published:** 2023-06-22

**Authors:** Tageldeen Saeed Tageldeen Ibrahim, Tilahun Seyoum Workneh

**Affiliations:** 1 Department of Agricultural Engineering, Faculty of Agriculture, University of Khartoum, Khartoum, Sudan; 2 School of Engineering, Discipline of Bioresources Engineering, University of KwaZulu-Natal, Pietermaritzburg, South Africa

**Keywords:** Environmental impact, Wastewater, Pollutants, Sugar industry, Sudan

## Abstract

The purpose of this study was to investigate the impact of Sudanese sugar manufacturing waste on the communities surrounding the industries. The study employed a cross-sectional survey in which 311 respondents living in factory areas. The selected sugar industries included Kenana, Guneid, Halfa, Sinnar, Assalaya, and White Nile. Data were analyzed using SPSS version 19. Descriptive statistics, nonparametric statistics, and logistic regression were employed. The results showed that wastewater discharge has a significant (*P* < 0.05) effect on community health. Respondents indicated that the waste creates an ideal environment for parasites to reproduce, off-odors to develop, and ultimately contamination of water. A multinomial logistic regression model showed that wastewater (i.e., off-odors and mosquitoes) have significant (*P* 0.05) influences on causing health risks (i.e., malaria) to people living around sugar factories. The study also revealed that the lack of sugar industry wastewater management has significantly affected crop and animal production. The suspended particles and bagasse fly were significant (*P* 0.05) in causing eye and respiratory system diseases in the region. Health services provided by the industries significantly (*P* = 0.05) impacted community satisfaction. In this regard, the study designed a framework for enhanced handling the industrial waste to be adopted by the Sudanese sugar industry decision-makers. A framework was developed to reduce the impact of waste to the lowest possible level by improving management strategies sufficiently to minimize its impact.

## Introduction

The environment is one of the main elements of individual and community health. The problem of pollution is considered to be far from satisfactory, especially in developing countries (Sahu, 2015). With the increase of rain-fed and irrigation agriculture, urbanization, and industrialization in Sudan, pollution is rapidly increasing (Alim, 2012: Pierre et al., 2016). However, the institutional and legislative frameworks are very limited and the pollution control measures need to be more effective. In a study conducted by Robert (2011) on the assessment of carbon emission reductions in Africa from improved waste management, it was concluded that there is a lack of knowledge about waste management practices in the country environmental management strategies were nonexistent in the industry management structure until the year 2000. It was reported by Alim (2012), in a master’s study that, the major challenges need to be identified, and an impact assessment needs to be undertaken to improve the operation of the older government-managed sugar industry. The sugar industry is one of the largest sources of industrial effluent and it generates a considerable amount of wastewater, including pollutants in the inform solids and gases (Sahu, 2015, 2018). The sugar industry in Sudan discharges untreated wastewater that contains pollutants that poison the watercourses (Alim, 2012; Anail et al., 2013). The volume of the discharged effluent varies from one factory to another, depending on its cane-crushing capacity (Sahu, 2015). The estimated daily discharge of wastewater for all sugar factories in Sudan is about 150,000 m^3^. Aisha (2007) funded a master study that, a small proportion of this wastewater is used for crop irrigation, but its impact on health is uncertain. A reasonable amount of waste, such as filter cakes and vinasse, also resulted from the manufacture of sugar (Oboody, 2016). These pollutants harm humans and the surrounding ecosystem (Oboody, 2016; Sahu, 2018), for example, the wastewater that is discharged into open fields has an impact on the environment and the community residing in the vicinity of the industries. Contamination such as acidification and heating of the river water could also cause health risks (Günter et al., 2007). This issue needs to be solved in Sudan. The environmental aspects must therefore be analyzed by identifying the impact of this effluent on the communities that live in the vicinity of selected sugar factories.

Bagasse is one of the waste products of the sugar industry, and it is used for steaming the boilers of factories (Sahu, 2018). It is combusted during the sugar manufacturing process and produces ash, which influences human health (Le Blond et al., 2017; Mohamed & Samah, 2011). Roughly 11,284 t of residual ash is produced annually in the Sudanese sugar factories (Cordiero et al., 2004). The impact of such pollutants needs to be studied and solutions need to be found; however, the authorities are concerned about the high cost of waste treatment (Oboody, 2016). Abid (2008) conducted a master study and concluded that there is a lack of sufficient data on waste treatment and its impact on the communities residing around these sugar factories, which makes it very difficult to identify the adverse effects of the waste and then find proper solutions.

Moreover, the sugar industry produces large amounts of gases, for example, carbon which influences the health of animals, plants, and humans. Dust storms that are produced during sugarcane harvesting influenced the communities residing around the factories in Sudan. Sugar factories are also a source of noise, due to the various operations and heavy machinery used during the production process (Abid, 2008). From an environmental perspective, the industry is facing problems related to pollutants because of mismanagement and industrial standardization (Sahu, 2018). However, no clear method has thus far examined the adverse effects of sugar manufacturing pollutants on the community residing around the selected sugar industries in Sudan. Therefore, it is necessary to study their impact on the environment and the community living in the vicinity of the factories.

Waste management strategies are significantly different among various countries, which is why achieving certain objectives remains a prominent issue. A well-designed framework will steer managers to address the waste issue in a cost-effective and timely manner, and it will spur the enhancement of existing plans or assist in the design of new ones (Davidson, 2011). Different techniques were developed to address the waste problem, such as integrated waste management (IWM), which combines methods, technologies, and management strategies to achieve particular goals. A systems analysis provides useful information for defining, evaluating, and adapting waste management systems (Pires et al., 2010). The sugar waste could also be used as a source of energy and as raw material for environmentally friendly products (Evgeniya et al., 2017). This study aims to evaluate the impact of waste on the environment and the communities surrounding the selected industries in Sudan. The study also aims to create a framework for the integral handling of industrial waste, to spur the decision-makers towards enhancing the environment of the sugar industry in Sudan.

## Research methods

### Study areas

**Fig. 1:**
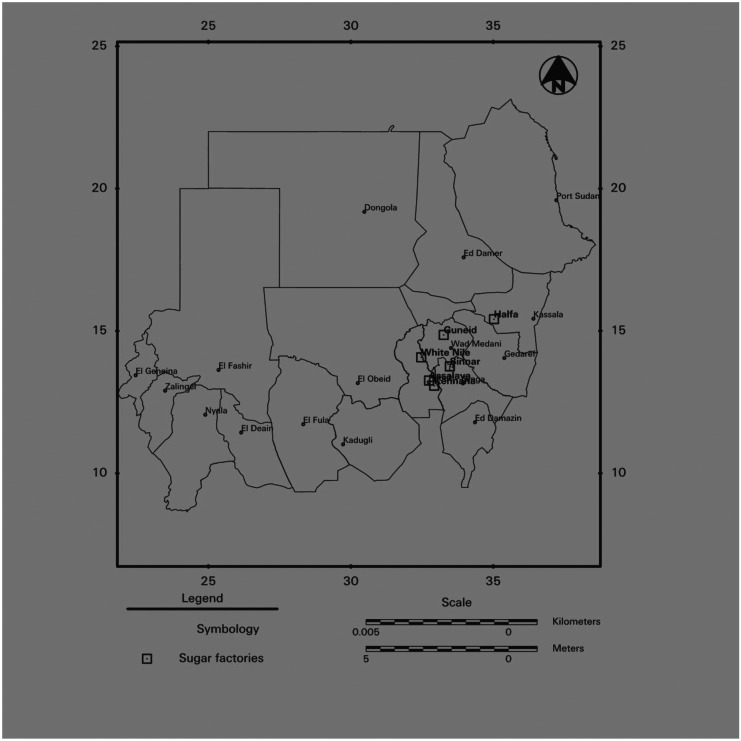
locations of the Sudanese sugar factories

This study was carried out in different locations around six sugar industries in Sudan. The selected sugar industries are Guneid, Halfa, Sennar, Assalaya, Kenana, and White Nile. Kenana (Fig. 1), White Nile, and Assalaya industries are from White Nile State. While Guneid is from Gazeira State, Sinnar is from Sinnar State and Halfa is from Kassala State (Fig. 1). Guneid is located between longitude and latitude; 14° 51.55 N 33° 15.62 E, Halfa location is roughly between 15° 28 N 35° 34.3 E, Sinnar 13° 45.3 N 33° 28.5 E, Assalaya 13° 15.434 N 32° 44.745 E, White Nile 14° 4.30 N 32° 28.21 E, and Kenana is between 13° 4.5 N 32° 55.6667 E. Figure 1 was designed by Mohammed (2022) from the department of remote sensing, University of Khartoum, Sudan.

### Data collection

This study is a cross-sectional survey approach and was conducted with self-administered semi-structured questionnaires (Mengistu et al., 2016). The questionnaire contained close-ended questions that were developed and pretested by experts to avoid ambiguity and refine categories. The pretesting was done by questioning about five to 10 persons from those non-targeted respondents. The questions were structured in a way that allows and considers very detailed insights. For example, the pollutants with their different types and their impact on the surrounding environment. The health service is presented by the authorities to the community living in the vicinity of the selected industries (Hind, 2015). Also, the effect of the factories’ intervention on people’s incomes and lifestyles was considered. Appendix A summarizes samples of the survey questions that were constructed in the questionnaire. The interviews were carried out during the period from January/2017 to March/2017. The questions used a three-point Likert scale for agreeing or disagreeing and neutral. The reason for choosing only three points was because of the simplicity of the majority of the community living in the vicinity of the industries areas. The researcher has comprehended that the answers to questions should be set most easily for the respondents. This method has enabled the researcher to collect clear-cut information from the respondents. The degree of complaints was set to four levels (i.e., no complaints, low, medium, and high) to measure the effectiveness of the stated pollutants on the surrounding community. The targeted population was the families who are living on campuses and villages close to the selected industries. The samples of this study comprised 377 respondents (families) selected randomly by lottery method from the selected residential areas around the industries. The number of samples has been determined by using Eq. (1) as it showed below (Taro, 1967). The questionnaire was divided into nine main questions.

1 $$n=\frac{N}{1+N{(e)}^{2}}$$

where: *n* = sample size, *N* = population size, *e* = 0.5, 0.3, 0.7

### Sampling techniques

The random sample technique was used for this study and the sample unit was the respondent’s family to be many representatives (Mengistua et al., 2016). Questionnaires were distributed randomly to the families who are living around within a diameter of 20 km of the selected industries. There were three targeted locations around each industry covered by about 21 questionnaires for each. The questionnaire was carried out to be filled out by all the family members. The main reason for selecting respondents settled around the industries was to allow very detailed insights and acquire clear-cut information about the raised issues in the questionnaire.

### Data analysis

The statistical package for social science (SPSS) was used to analyze the obtained data from the questionnaires. SPSS 19 is well-known computer software to supports statistical analysis of survey data. The descriptive statistics included percentages, means, standard deviation, and standard error. Nonparametric statistics encompassed the Mann–Whitney test and the chi-square test used to compare mean values of variables and to identify significant differences. For example, diseases caused by the pollutants were identified and the relation between the other parameters was found by the correlation coefficient test. Furthermore, multinomial logistic regression was employed to find out the important factors influencing the surrounding environment. Furthermore, multinomial logistic regression was employed to find out the important factors influencing the surrounding environment. Multinomial logistic regression is a probability estimation model used when the dependent variable is more than two categories (i.e., agree disagree and neutral) and the independent variable is categorical or continuous (John et al., 2017). Table 1 shows the dependent and independent variables associated with influencing the environment and their definitions.

**Table 1:** variables influencing the environment of the Sudanese sugar factories and their descriptions

Variable	Description	Type
Wastewater creates off-odor	Agree = 1, disagree = 2, and neutral = 3	Nominal
Wastewater creates mosquito		
Wastewater mixes with a water source		
*People’s health (i.e., malaria)		
Particulates contaminate the air		
Particulates pollute the flour and clothes		
Invisibility due to smoke clouds		
*Health risks to human	Stomach ache = 1, vomiting = 2, diarrhea = 3, and other = 4	Nominal
*The infection (eye disease, heart attack, respiratory disease, asthma, chronic bronchitis, and irregular heartbeat)	Yes = 1 and no = 2	Ordinal (binary)
*Disease cases among the animals		
*Death cases among the animals		
*Risk to crop		
*People complain	High = 1, medium = 2, low = 3, and no complain = 4	Ordinal

^*^Dependent variable

If *Y* is the dependent variable, it can take values of 1, 2, or 3.

*Yi* = 1 if the respondent agrees with the raised question

*Yi* = 2 if disagree

*Y*i = 3 if neutral

Hence, the multinomial logistic regression model for estimating the influence of wastewater on community health is as follows:

2 $$\mathit{ln}\left[\frac{{\rho }_{h}}{{\rho }_{j}}\right]={\beta }_{0h}+{\beta }_{1h}{x}_{1}+{\beta }_{2h}{x}_{2}+\cdots {+\beta }_{kh}{x}_{k}$$

where

*ln* = the log of the odd ratio

*P* = the probability of community health effect

*j* = the number of categories (3)

h = 1 to *j* -1

*β* = a constant

*β*_1_, *β*_2,_ and *β*_*k*_ = the estimated parameters corresponding to each predictor

*X*_1_, *X*_2_, and *X*_*k*_ = the explanatory variables (predictors)

*k* = the number of predictors

To compare the probability of one of the categories, odds ratios are all compared to the reference outcome by using the following equations:

3 $$\mathit{ln}\left[\frac{{\rho }_{1}}{{\rho }_{3}}\right]={\beta }_{01}+{\beta }_{11}{x}_{1}+{\beta }_{21}{x}_{2}+\cdots +{\beta }_{k1}{x}_{k}$$

4 $$\mathit{ln}\left[\frac{{\rho }_{2}}{{\rho }_{3}}\right]={\beta }_{02}+{\beta }_{12}{x}_{1}+{\beta }_{22}{x}_{2}+\dots +{\beta }_{k2}{x}_{k}$$

Equation 2 represents the probability of respondents agreeing (*P*_*1*_) with compared to neutral (*P*_3_) to the issue of health risks caused by wastewater. Equation 3 illustrates the comparison of the probability of disagree category (*P*_2_) to the neutral (*P*_3_) category (Grace-Martin, 2018).

## Results and discussion

### The effect of wastewater on people residing in the vicinity of the industries

**Fig. 2:**
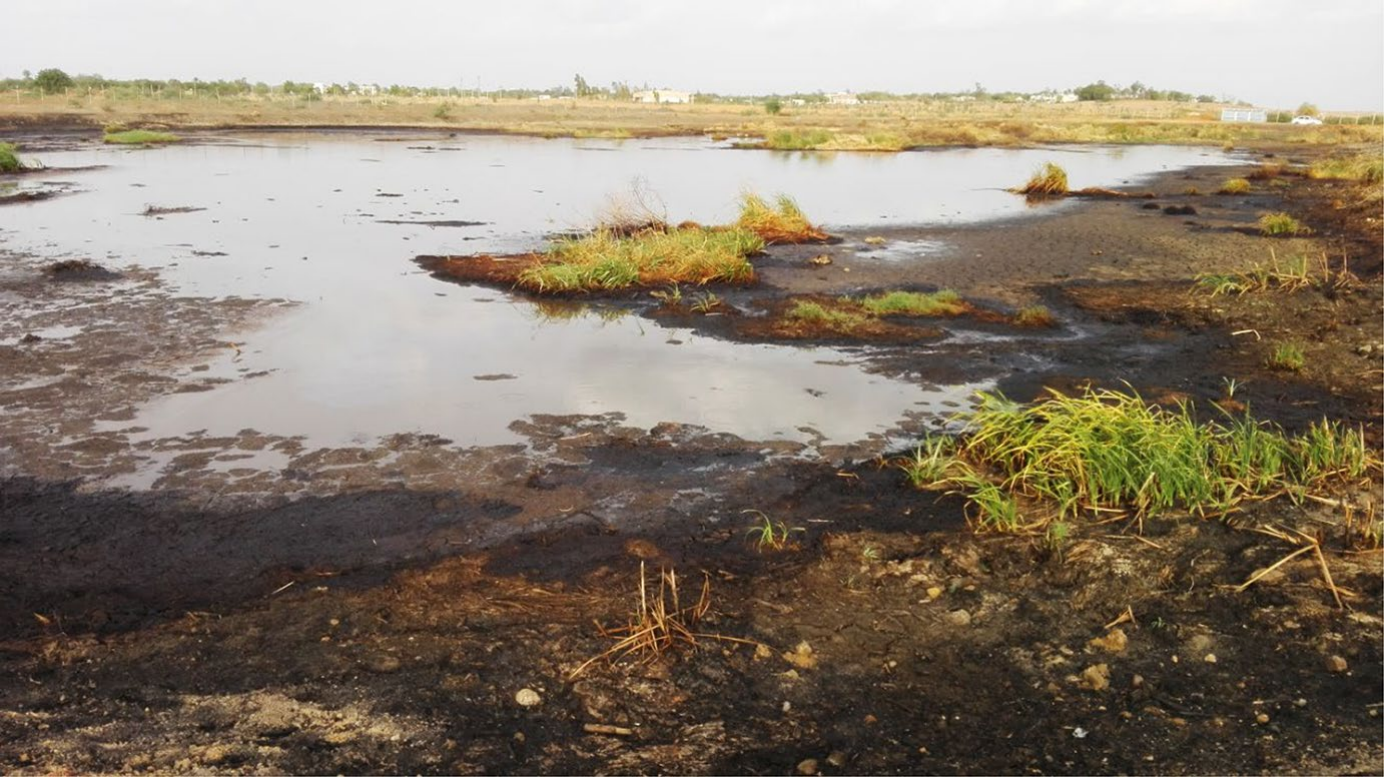
Wastewater creates parasites in the sugar industry

**Fig. 3:**
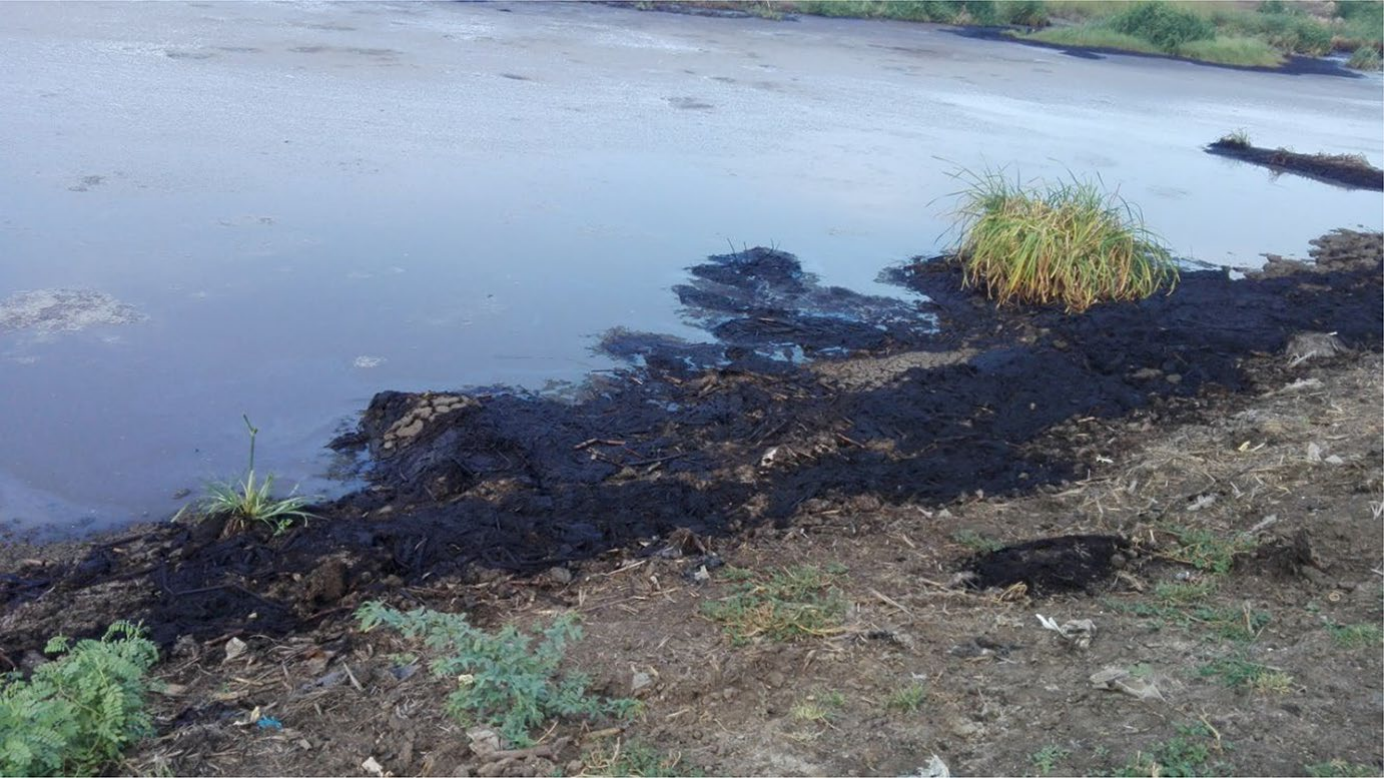
Wastewater released into open fields in the sugar industry

The descriptive statistics of the variables associated with the effect of wastewater on the surrounding community are displayed in Table 2. It was found that wastewater had a statistically significant (*P* < 0.05) effect on the mean differences in the creation of an off-odor, mosquitoes, as well as mixing with the water source and causing health risks (i.e., malaria). Figure 2 shows the wastewater creates a suitable environment for the reproduction of parasites in one of the selected sugar industries. The nonparametric statistics showed that the creation of parasites and an off-odor by wastewater, significantly (*P* < 0.01) influenced human health, whereas the wastewater was significantly (*P* < 0.05) responsible for contaminating the water sources used for drinking purposes. In a study conducted in Assalaya, water-related diseases (i.e., vomiting, diarrhea, and allergic) were observed among the community that used the surplus irrigation canals that were influenced by factory effluents (Ahmed et al., 2017). This may have been due to the lack of health awareness in the villages around the sugar industries. It might be also because most of the sugar industries in the country are located near the Nile River, which increases the chances of water-source contamination. Figure 3 shows the wastewater being released into open drains in one of the selected sugar factories. This was in agreement with the findings of Hind (2015), who concluded that the simple undeveloped lifestyle of the communities living around the factories may have endangered their health. It also concurred with the conclusion of Elhag (2010), who conducted a master’s study on cleaner production opportunities in the sugar industry in Sudan, and indicated that the lack of awareness of the impact of pollution is one of the problems phasing the Sudanese sugar industry.

**Table 2:** Descriptive statistics of the variables illustrating the environmental impact of sugar industry waste

Variables	***N***	Agree (%)	Disagree (%)	Neutral (%)	Mean	S.D	S.E	Sig
Wastewater creates off-odor	311	196 (63)	87 (28)	28 (9)	1.43	0.628	0.036	0.000
Wastewater creates mosquitoes	311	209 (67.2)	77 (24.8)	25 (8)	1.40	0.608	0.035	0.000
Wastewater contaminates water	311	121 (38.9)	160 (51.4)	30 (9.6)	1.44	0.634	0.036	0.000
*People’s health (malaria)	311	206 (66.2)	70 (22.5)	35 (11.3)	1.45	0.689	0.039	0.000
Particulates contaminate the air	305	260 (83.6)	34 (10.9)	11 (3.5)	1.18	0.47	0.027	0.000
Particulates dirty the floors and clothes	305	278 (89.4)	21 (6.8)	6 (1.9)	1.11	0.369	0.021	0.000
Invisibility due to smoke clouds	305	236 (75.9)	49 (15.8)	20 (6.4)	1.29	0.582	0.033	0.000
Loud sounds	305	204 (65.6)	70 (22.5)	31 (10)	1.43	0.671	0.038	0.000

	*N*	Stomach ache (%)	Vomiting (%)	Diarrhea (%)	Other (%)	Mean	S.D	S.E	Sig
*Health risks to human	108	38 (12.2)	3 (1)	54 (17.4)	13 (4.2)	2.39	1.09	0.105	0.000

* Infection:	*N*	Male (%)	Female (%)	Mean	S.D	S.E	Sig
Eye diseases	125	82 (26.4)	43 (13.8)	1.34	0.477	0.043	0.000
Heart attach	10	6 (1.9)	4 (1.3)	1.40	0.516	0.163	0.527
Respiratory diseases	62	40 (12.9)	22 (7.1)	1.35	0.482	0.061	0.022
Asthma	40	30 (9.6)	10 (3.2)	1.25	0.439	0.069	0.002
Chronic bronchitis	65	42 (13.5)	23 (7.4)	1.35	0.482	0.060	0.018
Irregular heartbeat	18	13 (4.2)	5 (1.6)	1.28	0.461	0.109	0.059

	*N*	Yes (%)	No (%)	Mean	S.D	S.E	Sig
*Diseases to animals	204	90 (28.9)	114 (36.7)	1.56	0.498	0.035	0.093
* Animal deaths	205	94 (30.2)	111 (35.7)	1.54	0.499	0.035	0.235
*Risk to crop	206	124 (39.9)	82 (26.4)	1.40	0.491	0.034	0.003

Variables	*N*	High (%)	Medium (%)	Low (%)	No (%)	Mean	S. D	S.E	Sig
Off-odor	309	137 (44.1)	60 (19.3)	42 (13.5)	70 (22.5)	2.15	1.212	0.069	0.000
Mosquitoes	309	194 (62.4)	61 (19.6)	36 (11.6)	18 (5.8)	1.61	0.908	0.052	0.000
Flies	309	148 (47.6)	83 (26.7)	48 (15.4)	30 (9.6)	1.87	1.005	0.057	0.000
Sugarcane burning particles	309	193 (62.1)	74 (23.8)	29 (9.3)	13 (4.2)	1.55	0.830	0.047	0.000
Bagasse particles	309	155 (49.8)	67 (21.5)	46 (14.8)	41 (13.2)	1.91	1.085	0.062	0.000

^*^Dependent variables

Moreover, the complaints of the respondents were found to have a significant (*P* < 0.05) effect on the mean differences in off-odor, mosquitos, and health risks caused by the wastewater. as it showed in Table 2. The surrounding communities suffered from pollutants, which may have harmed their health. The main reason for this might be the inadequate treatment of industrial effluent in the sugar industry. This result concurs with the findings of (Alnail et al., 2013; Mohamed et al., 2017; Pradeep & Omprakash, 2017). The studies indicated that the pollution of waterbodies was due to the disposal of waste by the sugar industry when it was discharged without being treated, which influenced the water quality and the ecological system. Oboody (2016) and Alnail et al. (2013) also observed the creation of insects, parasites, and off-odors during the stagnancy of the sugar industrial wastewater. Therefore, the authorities in the country should find an effective means for treating wastewater in the sugar industry.

### The impact of wastewater on animal production

One-hundred and forty-three respondents (43%) were involved in activities such as animal and crop production. Their water sources were the sugarcane irrigation drainage canals, wastewater streams, the Nile River, water tanks, Wells, Lakes, and sugarcane irrigation canals. Wastewater was used by 3.1% of respondents, compared to other available water sources. The water source was found to have statistically significant (*P* < 0.05) mean differences in animal production. Symptoms of sickness were observed in some animals, while animal deaths were found in 90 (30%) of 205 of the respondents, as shown in Table 2. In a study conducted in Pakistan, animals suffered different diseases and, in some cases, deaths were recorded due to the consumption of sugar industry effluents discharged into drains near the villages (Qureshi et al., 2015). The descriptive statistics revealed that the wastewater caused significant (*P* < 0.05) mean differences in animal production. The nonparametric statistics showed that wastewater had a significant (*P* < 0.05) effect on animal health, when compared with other water sources (i.e., cane irrigation canals), while, chemicals in the wastewater may also have caused health risks for the animals. These results concur with those of other researchers who have analyzed the wastewater from the sugar industry, both locally and globally, and found that the chemical oxygen demand (COD) was extremely high, resulting in the contamination of the water (Oboody, 2016; Reddy et al., 2014). The results also concur with the findings of Asmah (2017), who conducted a study on analyzing the wastewater resulting from sugar processing in the Kenana sugar industry lagoon. These results were in agreement with the findings of Mohamed et al. (2017), who concluded that the wastewater from the Assalaya sugar factory caused a threat to the agricultural environment and the superiority of its animals.

### The impact of wastewater on crop production

**Fig. 4:**
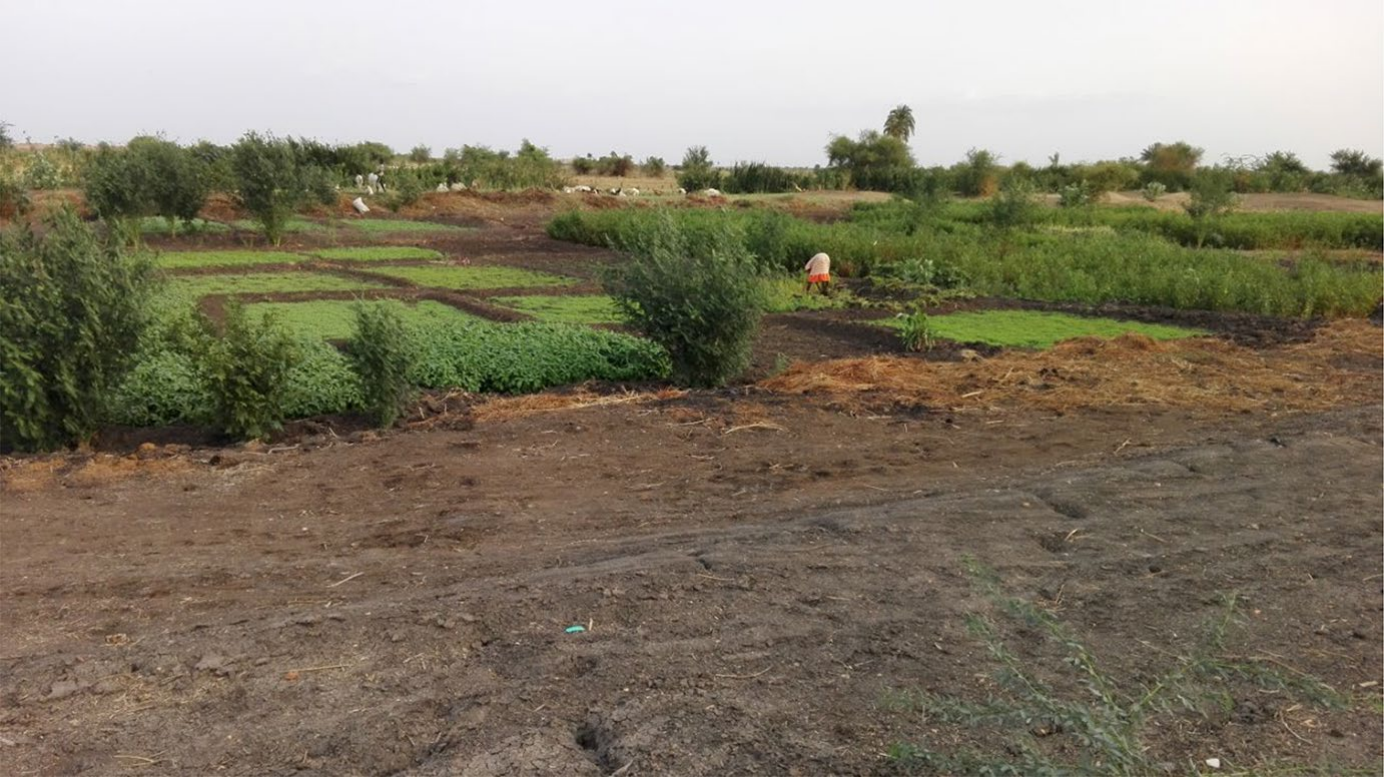
Crop and animal production influenced by wastewater in the sugar industry

Crops, like vegetables, cereals, and fruits were planted in small-scale farms (0.4 ha). Vegetables were the main cultivated crop (69%), compared to 28 and 3% which were under cereals and fruit trees, respectively. Figure 4 illustrates some crop that is produced by using wastewater in the Kenana sugar industry. Of the total 177 respondents, 44 (25%) used wastewater without pretreatments for crop irrigation, whereas 133 (75%) used sedimentation pans. Wastewater pre-treatment (i.e., sedimentation pan) was found to have a statistically significant (*P* < 0.01) effect on the mean differences in crop production. However, the nonparametric statistics revealed that there was an insignificant difference (*P* > 0.05) in the effect of using pre-treated wastewater for crop irrigation on human health, compared to the non-treated wastewater. There could be many reasons for the utilization of wastewater for crop irrigation. Firstly, it may due to the proximity of the streams to the fields, and secondly, it might also be due to the unavailability of an alternative water source for irrigation. This finding concurs with the findings of Saranraj and Stella (2014) who concluded that sugar mill effluent was used for plant irrigation in India, due to the lack of water sources. On the other hand, there is a consensus that wastewater is enriched with nutrient elements. The present finding showed that the health risks were not considered by producers, even though the wastewater had significant (*P* < 0.05) mean differences in causing risks to the crops. It was found that the consumption of vegetables irrigated with untreated wastewater significantly (*P* < 0.01) increased the susceptibility to infection and diseases (i.e., stomach aches and diarrhea). Aisha conducted a master’s study in 2007 on the impacts of sugar industry wastewater on some vegetable crop production and concluded that there was uncertainty about the healthy consumption of crops irrigated with effluent from the sugar industry. However, Kumar (2014) found that such effluent can be used for crop irrigation, under very restricted conditions, if appropriate dilution takes place. A risk may be caused by the accumulated chemicals, such as heavy metals, that will consequently influence human health. This is in agreement with the findings of researchers like Kumar (2014), Vinod (2014), Reddy et al. (2014), Sahu (2015), and Alnail et al. (2013), which indicated that untreated sugar industry effluents contained a significant proportion of chemicals, which contaminate the land, water, crops, and the air, which may negatively influence the quality of water used for drinking and irrigation purposes. Another study conducted in India found that the long-term usage of contaminated sugarcane irrigated with industrial effluents in rural areas results in a health risk (Bhawna et al., 2016).

### The impact of pollutants on the community surrounding the industries

**Fig. 5:**
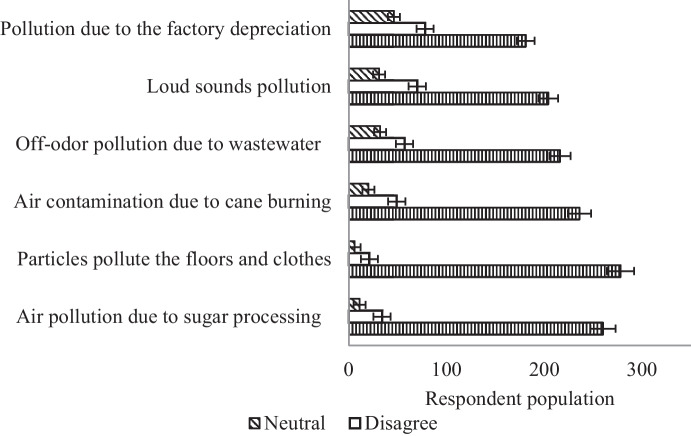
People sufferings from pollutants in their environment

**Fig. 6:**
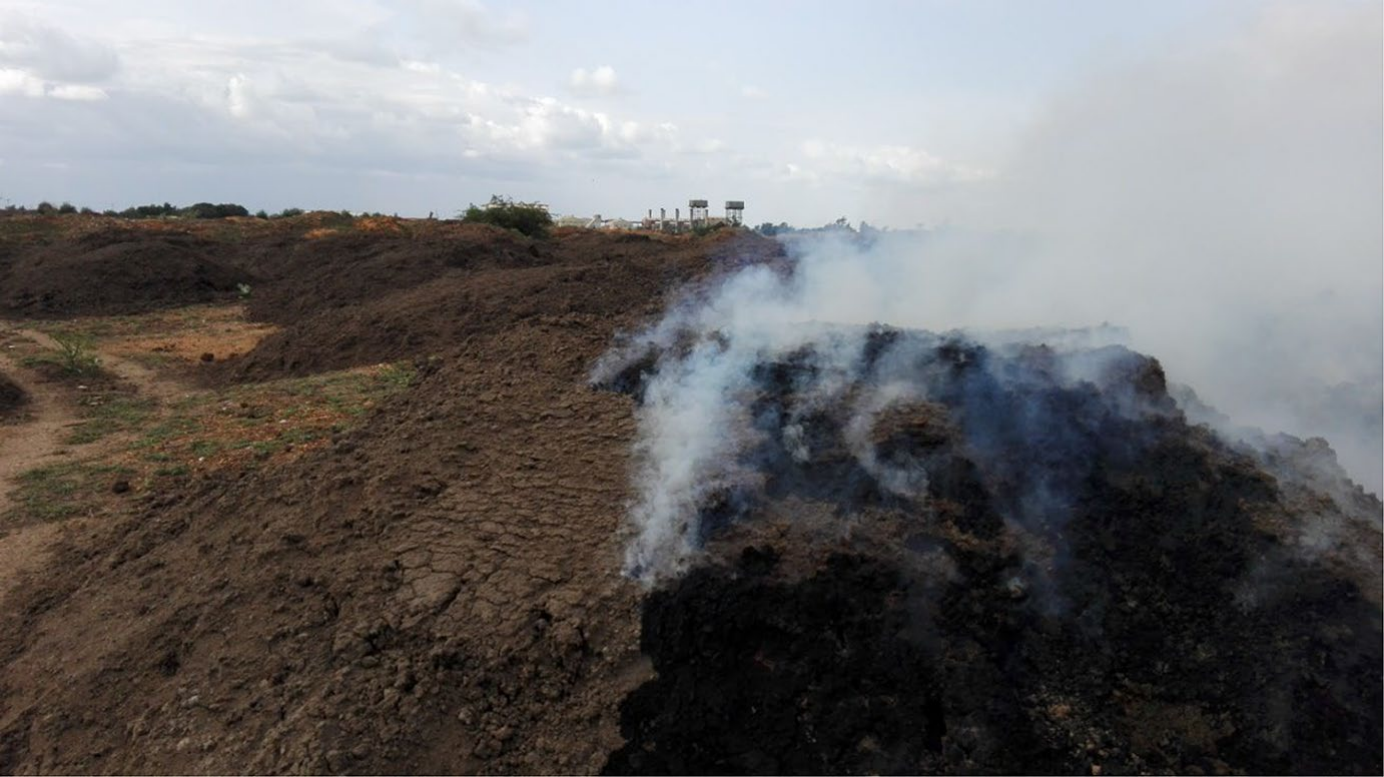
Emissions from bagasse and filter cakes in the Kenana industry

**Fig. 7:**
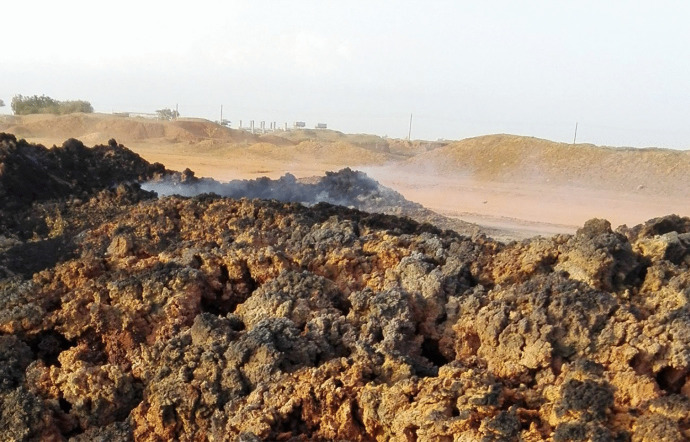
Bagasse and filter cake ash in the sugar industry

The sugar industry in Sudan has been found to release huge quantities of pollutants (i.e., organic particles, noises, and smoke clouds) during the processing operation. People suffer from the massive spread of organic pollutants and particles, as it showed in Fig. 5. The descriptive statistics have shown that the pollutants significantly (*P* < 0.01) contaminate the air and pollute the clothes and floors of the community surrounding the sugar industries. The nonparametric statistics revealed that the respondents suffered significantly (*P* < 0.05) from the suspended particles that resulted from sugarcane and bagasse burning. This could be because no pollution measures are taken by the sugar industry sector in the country. Figures 6 and 7 show the smoke and ash resulting from burnt bagasse and filter cake in the dumping area of one of the industries, which is emitted and could be drifted into the air. It was reported by TIFAC (2019) that the installation of air pollution control equipment would help to prevent pollutants (i.e., ash) from fully escaping into the atmosphere through the chimneys. Table 2 also shows that the loud sounds, due to the operations taking place during the sugar processing season, significantly (*P* < 0.05) influence the people living in the nearby villages. This might be due to the proximity to the factories (i.e., a distance of one km on average). Moreover, it was observed that a large number of pollutants were released from the chimneys of sugar factories. The nonparametric statistics showed that the depreciation of the factories as well as old-fashioned approaches practiced in the sugar industry in Sudan, significantly (*P* < 0.01) increased the suffering of the villages in the vicinity caused by an off-odor and parasites (i.e., mosquitoes and flies). This concurs with the findings of engineers during personal communication with Yosuf (2017), and a personal communication conducted with Yassir (2017), who stated that the publicly owned factories in the country had depreciated and that the technology had not been updated, which may affect the surrounding environment. This was in agreement with a report by TIFAC (2019), which stated that there was a reduction in visibility in the areas surrounding the sugar mills because of the massive release of pollutants into the air. The present findings indicated that there are still more aspects (i.e., noise pollution) that influenced the communities in the areas surrounding the factories. Although the effects of pollutants resulting from sugar manufacture are inevitable, Wada et al. (2017), stated that they could be minimized. For instance, villages should be kept far away from the industries areas, and the authorities should embark on a rehabilitation program to improve the performance of industrial chimneys, to reduce the effects of emissions.

### Health risk of pollutants on the community in the vicinity of the industries

The responses to the questions about their health were relatively low, compared to the total targeted population. Out of a total of 311 respondents, only 151 (48%) answered the questions relating to the health issue. This might firstly, be due to the sensitivity of the issue and to this particular society wanting to avoid conflict with the authorities. Secondly, it might be due to the lack of health awareness among the communities living in the vicinity of the industries. This concurs with the findings of Hind (2015) who highlighted the simplicity of the community surrounding the sugar factory in Kenana. This has made people reluctant to respond to questions dealing with both environmental and health issues. However, the authorities should take effective measures to deal with this issue transparently, to develop a viable solution for mitigating the health risks that may be caused by the industry pollutants.

**Fig. 8:**
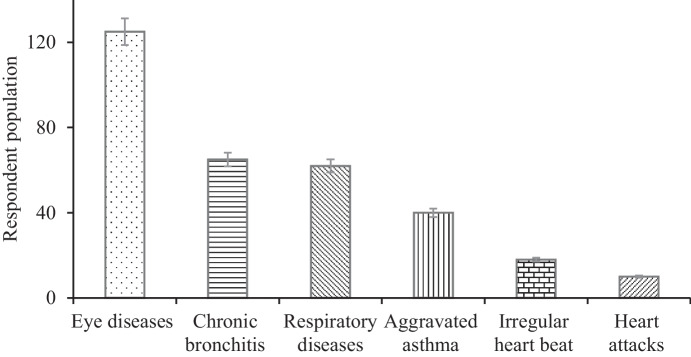
Diseases caused by sugar manufacturing waste

The pollutants caused diseases, such as eye allergies and infections, chronic bronchitis, respiratory infections, asthma, irregular heartbeat, and heart attacks for 125 (82.7%), 65 (43%), 62 (41%), 40 (26.5%), 18 (12%), and 10 (6.6%) of the respondents, respectively (Fig. 8). Eye diseases (i.e., allergies and infections) were found to have a highly significant (*P* < 0.01) mean difference in the health of the community. Table 2 also shows that the respiratory diseases (i.e., asthma and chronic bronchitis) were significantly (*P* < 0.05) widespread among the residents. Paula et al. (2017) found that people who resided close to the sugarcane-burning areas in Brazil were significantly susceptible to cardiovascular morbidity. The study estimated that the effect of exposure to air pollutants on people with cardiovascular disease was evidence of the health risks caused by sugar manufacturing pollutants. The present findings have revealed that the impact of sugar manufacturing pollutants on human health could cause a wide range of diseases for those who are living in the vicinity of the industries. Qureshi et al. (2015) reported that the waste discharged by sugar mills in Pakistan was found to cause asthma and various skin diseases, whereas in a study conducted in India, dizziness and physiological effects such as irritation in the eyes, nose, throat, and lungs were recorded in people living in areas surrounding the sugar mills (TIFAC, 2019).

### Industry participation in the community

**Fig. 9:**
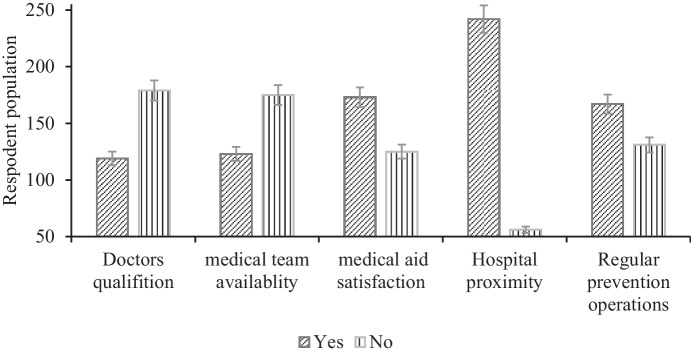
The satisfaction of the population with health services

It was found that the sugar industry in Sudan provides services to its communities (i.e., hospital accessibility, medical aid, the availability of qualified staff, and scheduled prevention rotations). The hospitals were found to have a statistically significant (*P* < 0.05) mean difference in their closeness to the villages, and the availability of doctors and medical teams in the hospitals significantly (*P* < 0.01) influenced the people’s satisfaction. The people were significantly (*P* < 0.05) satisfied with the regular prevention works (i.e., the spraying of pesticides against parasites) (Fig. 9). In a master study in South Africa conducted by Takalani in 2013 on social impact assessment of sugar production operations in South Africa: a social life cycle assessment perspective, some cases of medical shortages were in hospitals surrounding communities. It reported that there was dissatisfaction with the health services, due to shortages of doctors and the necessary medical equipment. This may be due to society not having a scientific background on what constitutes a healthy environment. It could be also because the authorities are ignorant about the issues of community health and their surrounding environment. The survey results showed that there were no centers for monitoring the effects of sugar manufacturing waste on human health. This concurs with a master’s study by Elhag (2010) that was conducted in the Halfa sugar factory, which concluded that authorities within the sugar industry were unaware of the impact of waste products on both the environment and human health.

### Issues that should be taken into consideration

**Fig. 10:**
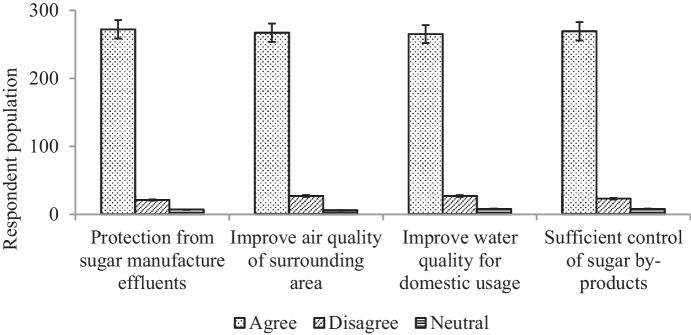
Response to the important issues that should be considered

It was revealed that serious issues were threatening both the environment and the health of the community surrounding the selected industries. Out of 300 respondents, the majority 268 (89%) agreed that the management was inefficient and that the communities were not protected against the waste from the sugar industry. People’s responses were found to have significant (*P* < 0.01) mean differences regarding the importance of protecting the community from the effluents, improving the air and water quality, as well as enhancing waste management and the surrounding environment (Fig. 10). This may be because the same old-fashioned practices have been followed since the inception of the majority of the Sudanese sugar industries. The conventional approach of sugar producers in Sudan seems to be to focus on the economic benefits, rather than on the environmental impacts. In a master’s study conducted by Elhag (2010), it was concluded that the sugar industry in Sudan was facing environmental problems due to its old follows. However, the authorities within the sugar industry should undertake effective strategies to decrease the environmental impact on the communities who live in the vicinity of the industry. Günter et al. (2007) reported that the sugar industries in Brazil are obliged to use waste-reducing technologies and water-cycling plants to keep the environment and water resources.

### Factors influencing the health of the community

The multinomial logistic regression model was conducted to identify the main factors influencing community health (i.e., malaria). The results of the analysis showed that the model fitted well with the observed data set. A statistically significant improvement in the fit of the model was found, where chi-square = 247.12, df = 32, and *p* < 0.001. The pseudo *R*-square (i.e., Cox and Snell *R*-squared, Nagelkerke *R*-squared, and McFadden *R*-square) values were 0.55, 0.66, and 0.46, respectively, which merely mimic the *R*-squared value in linear regression (Pallant, 2011). The value of McFadden *R*-square, which is often reported in research, was 0.46, which indicated a good fit for the data and supported the quality of the model.

Eight independent variables were included in the multinomial logistic regression test to define their effect on people’s health. Wastewater creates an off-odor, as well as the breeding of mosquitoes, which significantly (*P* < 0.05), influenced the health of the people residing in the vicinity of the sugar factories, by causing diseases (i.e., malaria). The distance of wastewater streams from the villages, the flow season, the mixing of waste with the water source, water source contamination, the creation of swamps, and the breeding of flies, were found to be statistically insignificant (*P* > 0.05) predictors in Table 3. The results revealed that untreated wastewater has encouraged the creation of off-odors and parasites (i.e., mosquitoes). Hence, it critically influences the surrounding environment and people’s health. Many studies indicated the relationship between the untreated wastewater of the sugar industry and the breeding of parasites (i.e., mosquitoes), which cause various diseases (Ahmed et al., 2017; Mohamed et al., 2017; Sahu, 2019). Therefore, sugar industrial wastewater has a major effect on the surrounding community and areas, unless it is treated.

**Table 3:** Multinomial logistic regression model results for the effect of wastewater on community health

I have had malaria several times	Parameter estimate	Standard error	Wald	*P*-value	Odds ratio
Wastewater stream is close (agree = 1)	0.528	1.419	0.139	0.710	1.696
Wastewater flows all the year (agree = 1)	1.033	0.956	1.168	0.280	2.810
Wastewater mixed with water body (agree = 1)	1.664	1.036	2.580	0.108	5.280
Wastewater contaminates water bodies (agree = 1)	1.118	0.972	1.323	0.250	3.058
Wastewater creates swamps (agree = 1)	0.085	0.898	0.009	0.925	1.089
Wastewater creates an off-odor (agree = 1)	0.436	0.869	0.251	0.616	1.546
Wastewater creates flies (agree = 1)	1.503	0.713	4.449	0.035*	4.495
Wastewater creates mosquitoes (agree = 1)	2.534	0.813	9.712	0.002**	12.61
Constant	− 3.911	1.603	5.950	0.015	0.021
Wastewater stream is close (disagree = 2)	− 1.271	1.499	0.719	0.397	0.281
Wastewater flow all the year (disagree = 2)	0.630	1.343	0.220	0.639	1.877
Wastewater mixed with water body (disagree = 2)	2.074	1.242	2.790	0.095	7.956
Wastewater contaminates water bodies (disagree = 2)	− 0.653	1.226	0.284	0.594	0.520
Wastewater creates swamps (disagree = 2)	− 0.834	1.114	0.560	0.454	0.434
Wastewater creates an off-odor (disagree = 2)	1.131	1.150	0.969	0.325	3.100
Wastewater creates flies (disagree = 2)	0.862	0.986	0.764	0.382	2.367
Wastewater creates mosquitoes (disagree = 2)	1.094	1.058	1.069	0.301	2.986
Constant	− 2.799	1.677	2.786	0.095	0.061

*and **Significant at *P* < 0.01 and *P* < 0.05, respectively. − 2 log likelihood = 221.351; Chi-square = 247.117 and *p* = 0.000. Pseudo *R*-square (Cox and Snell, Nagelkerke and McFadden) = 0.548, 0.669, and 0.465, respectively

### Wastewater is responsible for flies

The breeding of flies caused by the sugar industry wastewater was found to have a positive and statistically significant (*P* < 0.05) relationship with the endangerment of community health. The breeding of flies is more likely to influence the health of the people surrounding the sugar factories. The fact that more flies are created by the wastewater is more likely to fall under the “Agree” category than the “Disagree” or “Natural” categories. The probability of respondents agreeing to the issue of health risks appears more likely to increase by a factor of 4.5 as the level of flies increases. This seems to indicate that the wastewater of the sugar industry in Sudan is discharged without being treated, which mediates the reproduction of parasites. This result concurs with the findings of studies conducted in Sudan by Mohamed et al. (2017) and Ahmed et al. (2017) on the analysis of the wastewater of the Assalaya sugar factory. This result also agreed with the findings of a study conducted in Ethiopia on the treatment of sugar industry wastewater with ferrous material (Sahu, 2019).

### Wastewater is responsible for mosquitoes

Wastewater from the sugar industry was a positive predictor and a highly significant (*P* < 0.01) indicator for the breeding of mosquitoes and influencing the health of the surrounding community. The probability of the reproduction of mosquitoes in the sugar manufacture wastewater was more likely to be supported by the respondents than to fall into other categories (i.e., disagree and neutral). This means that the more wastewater is created, the more median of diseases (i.e., caused by mosquitoes) is created, which increases the probability of endangering the health of the surrounding community by a factor of 12.6. These results revealed that the sugar industry wastewater in Sudan is the main contributor to the breeding of mosquitoes. This concurs with a study in which the untreated sugar industry wastewater in Ethiopia was found to be a source of mosquitoes (Sahu, 2019).

### A prospective framework for handling the sugar manufacturing waste in Sudan

The communities surrounding the selected sugar industries in Sudan are facing unavoidable contamination. These contaminants could be minimized to the lowest levels by making a sustained effort to use efficient tools to manage the waste, thus mitigating the environmental impact. This study has therefore designed an integrated framework to conserve the bio-network of the sugar industry in the country. The framework is based on collaborative efforts between the sugar industry and the surrounding communities to improve waste management.

**Fig. 11:**
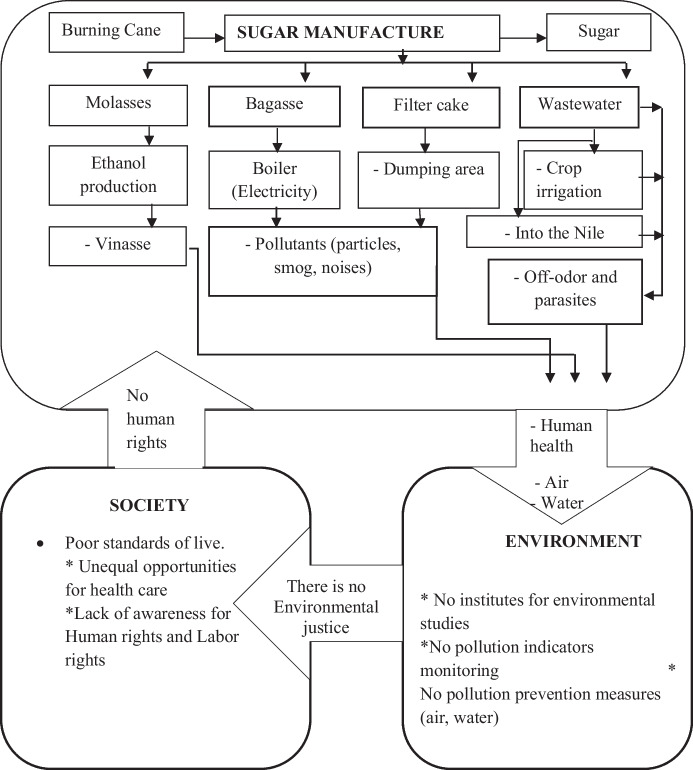
The current sugar waste management system

The concept of this framework depends on the industrial ecology, and it focuses on integrating and adapting technologies to sustain the improved management of sugar manufacturing waste (Davidson, 2011). The prospective industrial waste handling framework for the selected sugar industries aims to do the following: (1) maximize the reuse and recycling of sugar industry waste, (2) support decision-makers in achieving sustainable sugar production, and (3) achieve zero waste for the Sudanese sugar industry. These goals can be achieved by introducing new technologies which can transform the raw materials of sugar manufacturing waste into eco-friendly products. For instance, the building of a wastewater treatment plant as well as the idea of green harvesting of sugarcane will help to minimize the impact of pollution on the people that live in the vicinity of the industry. The existing practices that are used to treat sugar by-products and waste are not environmentally friendly. The surplus bagasse, filter cakes, wastewater, and vinasse are improperly managed. The filter cake, bagasse ash, and wastewater produce pollutants such as suspended particles, off-odors, and parasites. The pollutants are a health risk to humans and animals in the surrounding areas (Fig. 11). The authorities within the industry need to collaborate on the environmental aspects and the protection of society, to maintain a sustainable sugar industry bio-network in Sudan.

**Fig. 12:**
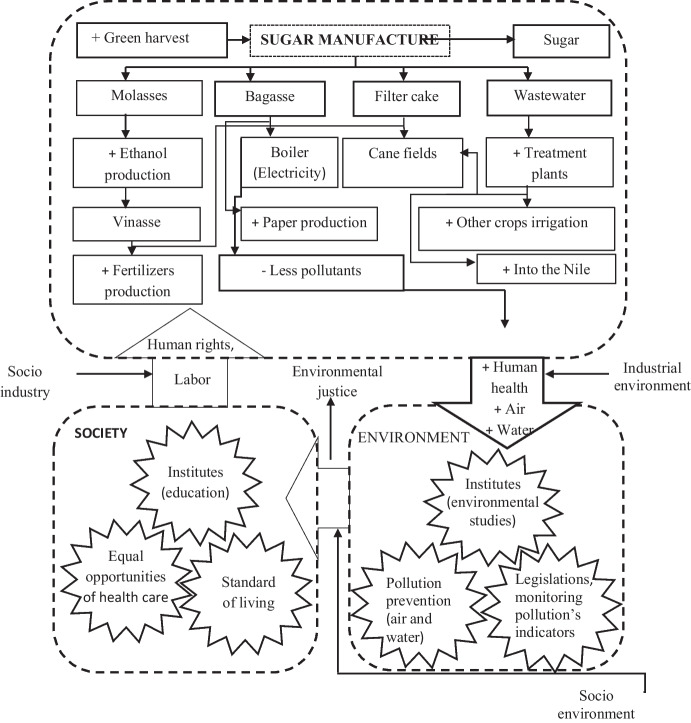
The prospective framework for the sugar waste management

The prospective strategy is to target specific by-products and waste to use them as raw materials for producing eco-friendly products (Fig. 12). The surplus bagasse could be used to produce paper. The wastewater could be recycled and reused, (Evgeniya et al., 2017; Prado et al., 2013). A Ph.D. study conducted in Egypt by Nakhla (2014) concluded that vinasse with filter cakes could be used to produce fertilizers, such as potassium and phosphate. The efficiency of on-site treatment plants could also be increased by initiating a wastewater recycling program (Oboody, 2016). Moreover, effective bodies must be sustained to work integrally with industrial, social, and environmental sides to conserve the bio-network of the sugar industry. Institutions should be created that is responsible for the implementation of extensive environmental, pollution, and waste management legislation. It is essential to collect information about pollution and waste monitoring, to implement pollution reduction measures. Sharing information is an important element for creating awareness about the effects of waste on human health. This framework is the first of its kind for the Sudanese sugar factories. The strategy aims at steering the decision-makers to reduce the environmental impact of sugar processing waste and pollutants. This will enable gaining a better understanding of the relationship between pollution, waste management, and healthy life for the surrounding community.where: [ −] = negative impact on the environment, [ +] = positive impact on the environment

## Conclusion

It has been identified that the Sudanese sugar industry’s waste disposal is causing problems for the surrounding communities and the environment. There have been significant (*P* 0.05) off-odors and mosquitoes created by the wastewater, which has been mixed with the water sources, which poses a health risk. Crop and animal production has also been significantly affected (*P* 0.05). Several diseases and deaths have been observed in animals and plants that utilize wastewater. Sugar industry pollutants (such as suspended particles and smoke clouds) have significantly caused eye and respiratory diseases (*P* = 0.05). The majority of respondents (85%) agreed that sugar manufacturing waste needs to be effectively managed and that the quality of water and air needs to be improved. Nevertheless, people’s satisfaction with doctors and medical teams in hospitals was significantly influenced (*P* 0.01). As a result, major reforms are needed to manage sugar manufacturing waste and eliminate its environmental impact. This study designed a framework for enhancing the handling of sugar industrial waste. The framework will positively affect the environment of the community residing in the vicinity of sugar factories. It will spur decision-makers to enhance the bio-network of the sugar industry in the country. Nonetheless, basic information is needed to better understand the health risks associated with the waste created by the industry.

## Data Availability

Data and materials of this study are available if needed for further review.
